# Carnation-like Morphology of BiVO_4_-7 Enables Sensitive Photoelectrochemical Determination of Cr(VI) in the Food and Environment

**DOI:** 10.3390/bios12020130

**Published:** 2022-02-19

**Authors:** Wenqin Wu, Zhao Tan, Xiao Chen, Xiaomei Chen, Ling Cheng, Huimin Wu, Peiwu Li, Zhaowei Zhang

**Affiliations:** 1 Key Laboratory of Detection for Mycotoxins, Ministry of Agriculture and Rural Affairs, National Reference Lab for Biotoxin Test, Oil Crops Research Institute of the Chinese Academy of Agricultural Sciences, Wuhan 430062, China; wuwenqin@caas.cn (W.W.); 13476831558@163.com (Z.T.); 202021106010763@stu.hubu.edu.cn (X.C.); chenxiaomei_200870@126.com (X.C.); chengling@caas.cn (L.C.); peiwuli@oilcrops.cn (P.L.); 2 Key Laboratory of Biology and Genetic Improvement of Oil Crops, Ministry of Agriculture and Rural Affairs, Oil Crops Research Institute of the Chinese Academy of Agricultural Sciences, Wuhan 430062, China; 3 College of Chemistry and Chemical Engineering, Hubei University, Wuhan 430062, China; whm267@hubu.edu.cn

**Keywords:** photoelectrochemistry, hexavalent chromium, bismuth vanadate, food safety, environmental monitoring

## Abstract

Hexavalent chromium, namely, Cr(VI), is a significant threat to ecological and food safety. Current detection methods are not sensitive to Cr(VI). A photoelectrochemical (PEC) sensor based on bismuth vanadate (BiVO_4_) was developed for sensitive detection of Cr(VI). First, BiVO_4_-X (X: the pH of the reaction precursor solution) was synthesized using a facile surfactant-free hydrothermal method. The BiVO_4_-X morphology was well controlled according to pH values, showing rock-like (X = 1), wrinkled bark-like (X = 4), carnation-like (X = 7), and the collapsed sheet-like morphologies (X = 9, 12). BiVO_4_-7 exhibited excellent photoelectric performance due to a proper band structure under visible light and a large specific surface area. Then, BiVO_4_-7 was used to construct a PEC sensor to detect Cr(VI), which was demonstrated to have a low detection limit (10 nM) and wide detection range (2–210 μM). The BiVO_4_-7 PEC sensor had a stable output signal, as well as excellent reproducibility, repeatability, and selectivity. We used the BiVO_4_-7 PEC sensor to detect Cr(VI) in real environmental and food samples, resulting in a satisfactory recovery of 90.3–103.0%, as determined by comparison with results obtained using a spectrophotometric method. The BiVO_4_-7 PEC sensor is promising for practical application to heavy metal detection in the food and environment.

## 1. Introduction

The rapid development of industrial manufacturing has made chromium contamination an increasingly significant focus in the environmental monitoring and food industries [1,2,3]. With a group I classification from the International Agency for Research on Cancer, hexavalent chromium (Cr(VI)) can cause genotoxic tumors, genetic defects, asthma, and allergies and harm the environment by accumulating in the ambient environment and food chains [4,5,6,7,8,9]. A sensitive detection method needs to be developed to trace Cr(VI) in the environment and food [10]. Current detection methods include atomic absorption spectrometry [11], fluorescence spectroscopy [12], high-performance liquid chromatography-inductively coupled plasma-mass spectrometry [13], and spectrophotometric methods [14], all of which have considerable sensitivity and accuracy but involve complex and laborious processes, expensive equipment, and dedicated operators [11,12,13].

A promising technique is the use of a photoelectrochemical (PEC) sensor, which offers the advantages of being low cost, requiring simple equipment that is easy to operate, and a low background signal [15,16,17,18,19]. However, a remaining challenge is to improve PEC detection sensitivity, which can be addressed by two strategies. One is surface modification via nanomaterials, such as BiPO_4_/BiOI [20], PbS [21], and TiO_2_ [22], for Cr(VI) detection in water samples [21,22]. Among various nanomaterials, scheelite monoclinic BiVO_4_ is one of the most promising visible-light-responsive electrode materials due to a wide bandgap, excellent stability, and low toxicity [23] that is widely used in optoelectronics research [24] and a potential nanomaterial for photoelectric detection of the heavy metal Cr(VI). However, bare BiVO_4_ has a low electron mobility, rapid photoelectric carrier recombination, and poor adsorption performance, resulting in low photoelectric catalytic performance [25]. Many strategies have been used to overcome these problems, including controlling the morphology [26], doping with metallic or nonmetallic elements [27], and coupling with multiple semiconductors to construct heterojunctions [28]. An alternative strategy, especially for Cr(VI) detection in food and soil samples, is to reduce the sample matrix effect by suitable sample preparation. A water sample is a typical matrix for Cr(VI) detection by a PEC sensor. Sample preparation mainly includes extraction by acid and alkaline solutions. However, sample preparation for Cr(VI) detection in solid agricultural products and food samples is more complex, and an extraction procedure is required to meet recovery requirements.

Odecyl benzene sulfonate (SDBS), polyvinyl pyrrolidone (PVP), cetyltrimethylammonium bromide (CTAB), dodecylamine (DA), oleylamine (OL), and oleic acid (OA) are usually used as surfactants to control the growth of the special shapes nanomaterials by adsorbing on the surface of BiVO_4_ nanoparticles. However, adding surfactants makes the operation more complicated. In this study, a hydrothermal method without surfactants (SDBS, CTAB, PVP, DA, OL, OA, etc.) was used to synthesize BiVO_4_ with a controlled morphology. Fine optimization of the pH used in the synthesis of BiVO_4_ resulted in a carnation-like morphology and excellent photoelectric properties. Then, a PEC sensor was developed, and the sensor stability, repeatability, and selectivity during application were evaluated. The PEC sensor based on the optimized BiVO_4_ with a carnation-like morphology was used for Cr(VI) detection in soil, rice, peanut, and water. A simple sample preparation protocol was developed to reduce the complex matrix effect. This PEC sensor can be extensively applied to monitor environmental and food safety.

## 2. Experiments

### 2.1. Chemicals and Reagents

The chemicals and reagents used in this study are listed in the Supplementary Material.

### 2.2. Synthesis and Characterization of BiVO_4_

A total of 15 milliliters of 5 mM Bi(NO_3_)_3_∙5H_2_O, 15 mL of 5 mM NH_4_VO_3_, and 5 mL CH_3_COOH were mixed for 30 min at room temperature. The final pH of the solution was adjusted to 1, 4, 7, 9, or 12 by adding NaOH (1 M), and the solution was heated to 100 °C for 20 h. The solution was cooled, washed with ethanol and water three times, and dried for 12 h at 80 °C. Finally, the powder samples were calcined at 350 °C for 2 h in a muffle furnace to obtain final samples of BiVO_4_-X (X = 1, 4, 7, 9, and 12, where X is the pH of the precursor solution). The crystal structure, photoelectric performance, morphology, valence state, and PEC mechanism of the synthesized materials were characterized via X-ray diffraction (XRD), electrochemical impedance spectroscopy (EIS), scanning electron microscopy (SEM), X-ray photoelectron spectroscopy (XPS), and UV-Vis diffuse reflection spectroscopy (UV-Vis DRS), and the results are provided in the Supporting Materials. Details of the instruments used are also provided in the Supplementary Materials.

### 2.3. PEC Sensor Preparation

A bare indium tin oxide (ITO) electrode (length: 2 cm, width: 1 cm) was ultrasonicated in acetone, ethanol, and ultrapure water for 10, 10, and 15 min, respectively. Then, 3 mg of BiVO_4_-X were dispersed in 0.2 mL of ethanol containing 0.3 mL of chitosan (0.5%), and the dispersion was ultrasonicated for 20 min. Finally, 30 μL of the BiVO_4_-X dispersion was coated on a treated ITO electrode before use.

### 2.4. Procedure for Using the PEC Sensor to Detect Cr(VI)

The BiVO_4_-based PEC apparatus is shown in the Supplementary Materials (Figure S1). After Cr(VI) was added to an electrolyte (0.1 M NaSO_4_), we immersed the sensor in the solution, and an electrochemical workstation was used to record the current signal with or without light. A correlation curve between the photocurrent signal and the corresponding Cr(VI) concentration was constructed and used to calculate the detection limit and range.

### 2.5. PEC Sensor Evaluation

A calibration curve was developed to calculate the LOD and linear range. A series of Cr(VI) standard solutions (2, 4, 6, 8, 10, 20, 30, 40, 50, 70, 90, 130, 190, and 210 μM) were used to establish standard curves for Cr(VI). Each data point in the standard curve was the result of three measurements. LODs were calculated using LOD = X + 3SD, where X is the average concentration determined by 21 repeated experiments on blank samples, and SD is the corresponding standard deviation. Spiked experiments were performed to evaluate the PEC sensor performance in terms of repeatability, reproducibility, stability, selectivity, and applicability to real samples. The repeatability was evaluated through 20 consecutive tests on 50 μM Cr(VI) using looped on-and-off light switching. Nine electrodes were tested to assess the sensor reproducibility for detection of 50 μM Cr(VI). The sensor stability was evaluated by comparing the photocurrent after 33 days with the initial photocurrent. The sensor selectivity was investigated by adding common ions Cl^−^, NO^3−^, Fe^3+^, Cu^2+^, Co^2+^, Zn^2+^, and Na^+^ to a 10 μM Cr(VI) solution. A real sample analysis was conducted on spiked samples of real peanut, rice, soil, and tap water samples (spiking concentrations: 10 and 100 μM Cr). Recoveries were calculated by comparing the results obtained using the PEC sensor and flame atomic absorption spectrometry.

### 2.6. Sample Pretreatment

The samples were pretreated following a certified method (Chinese Environmental Standard: HJ 1082–2019). A 5.0 g solid sample (e.g., soil, rice, or peanut) was extracted using a 50.0 mL mixed solution containing 0.28 M Na_2_CO_3_ and 0.5 M NaOH, to which 0.08 M MgCl_2_ and 0.5 mL of phosphate buffer solution (0.36 M K_2_HPO_4_ and 0.57 M KH_2_PO_4_, pH: 7) were added. The final pH of the filtrate was adjusted to 7.5 before use. The final sample filtrate was added to an electrolyte (0.1 M NaSO_4_), and we immersed the PEC sensor in the solution to detect Cr(VI) in real samples.

## 3. Results and Discussion

### 3.1. Characterization of BiVO_4_-X

**Figure 1:**
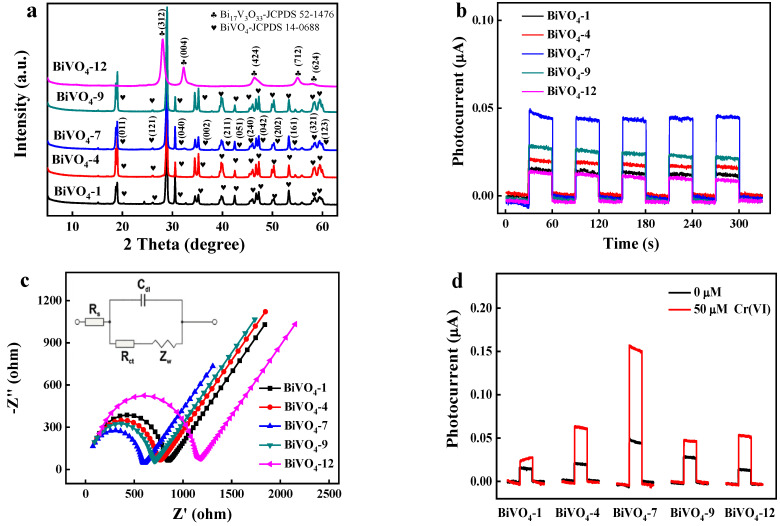
(**a**) XRD spectra of BiVO_4_-X (X = 1, 4, 7, 9, and 12); (**b**) photocurrent responses of BiVO_4_-X (X = 1, 4, 7, 9, and 12) in 0.1 M NaSO_4_; (**c**) EIS spectra of BiVO_4_-X (X = 1, 4, 7, 9, and 12) in 0.1 M NaSO_4_, where the frequency (Hz) ranges from 1 to 10^6^, and the inset shows the Randles equivalent circuit diagram; (**d**) photocurrent responses of BiVO_4_-X (X = 1, 4, 7, 9, and 12) (Cr(VI): 0 and 50 μM).

BiVO_4_-X (X = 1, 4, 7, 9, and 12) was synthesized without the use of surfactants. The XRD spectra of BiVO_4_-X (X = 1, 4, 7, 9, and 12) are shown in Figure 1a. BiVO_4_-7 spectrum exhibited peaks at 15.14°, 18.99°, 28.95°, 30.55°, 34.50°, 35.22°, 39.78°, 42.46°, 46.71°, 47.31°, 50.31°, 53.31°, and 58.53°, corresponding to diffraction from the (020), (011), (121), (040), (200), (002), (211), (051), (240), (042), (202), (161), and (321) crystallographic planes of monoclinic BiVO_4_ (JCPDS 14-0688). We found that BiVO_4_-1, BiVO_4_-4, and BiVO_4_-7 had the same crystal form, whereas BiVO_4_-9 and BiVO_4_-12 contained Bi_17_V_3_O_33_ (JCPDS 52-1476) due to vanadate hydrolysis.

The photoelectric performance of BiVO_4_-X was preliminarily evaluated via a photoelectric response experiment (Figure 1b) and an EIS test (Figure 1c). A lamp with an irradiation wavelength of 420 nm was switched on and off at 30 s intervals. The highest photocurrent in a 0.1 M NaSO_4_ blank electrolyte for BiVO_4_-X (X: 1, 4, 7, 9, and 12), therefore the highest photoelectric performance was obtained for BiVO_4_-7 (Figure 1b), and the photoelectric performance of the BiVO_4_-7 modified ITO sensor was 500 times higher than that of blank ITO substrates (Supplementary Material S2). The electrochemical performance of BiVO_4_-X was determined by EIS (Figure 1c). The semicircle diameter is a measure of the charge transfer resistance (Rct). The Rcts of BiVO_4_-X (1, 4, 7, 9, and 12) were 767, 690, 551, 644, and 1041 Ω, respectively, where BiVO_4_-7 exhibited the smallest Rct. BiVO_4_-7 exhibited the highest electrochemical performance, both in terms of photoelectric performance and EIS results. A BiVO_4_-X-based PEC sensor was used to detect Cr(VI), and the photocurrent responses of BiVO_4_-X (X: 1, 4, 7, 9, and 12) were recorded (Figure 1d). Among the BiVO_4_-X (X: 1, 4, 7, 9, and 12) detectors in the presence of 50 μM Cr(VI), BiVO_4_-7 showed the maximum photocurrent change and therefore, the highest photoelectric performance for detecting Cr(VI).

**Figure 2:**
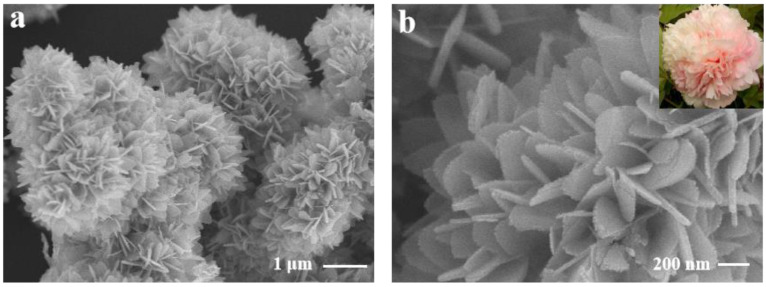
(**a**) SEM image of BiVO_4_-7 and (**b**) corresponding high-magnification image.

The SEM results showed that fine pH adjustment produced a diversity of morphologies for BiVO_4_-X (X = 1, 4, 7, 9, and 12). BiVO_4_-1 exhibited an irregular rock-like morphology (Figure S2a,b), and BiVO_4_-4 had the appearance of wrinkled bark (Figure S2c,d). Under neutral conditions, BiVO_4_-7 (Figure 2a,b) had a carnation-like structure assembled from regular sheets. Increasing the pH during BiVO_4_-X synthesis caused the sheet-like assembly structure to collapse (Figure S2e–h). Combining this result with the photocurrent responses of BiVO_4_-X showed that the largest current was obtained for the carnation-like morphology due to its uniqueness and increased surface area.

**Figure 3:**
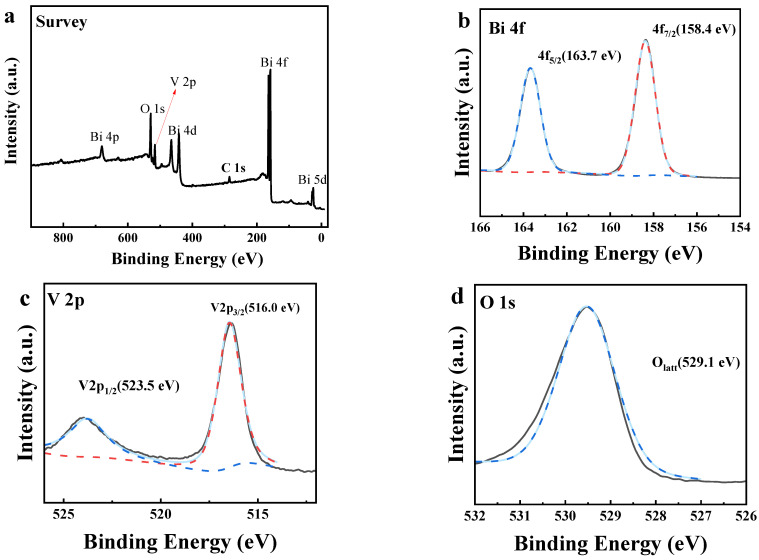
(**a**) XPS survey spectrum of BiVO_4_-7 and the corresponding high-resolution XPS spectra of (**b**) Bi 4f, (**c**) V 2p, and (**d**) O 1s.

The valence state and surface chemical composition of BiVO_4_-7 were characterized by XPS. The survey spectrum confirmed the presence of V, Bi, and O in BiVO_4_-7 (Figure 3a). The peaks at 163.7 and 158.4 eV were attributed to the spin-orbit splitting of Bi 4f_5/2_ and Bi 4f_7/2_, respectively (Figure 3b). The 5.3 eV spacing between the Bi 4f_5/2_ and Bi 4f_7/2_ peaks indicated a +3 valence state of Bi for an isotype heterojunction sample, as has been previously reported [29].

The two characteristic peaks at 523.5 eV and 516.0 eV correspond to V 2p1/2 and V 2p3/2, respectively (Figure 3c). The 7.5 eV spacing between the peaks of 2p1/2 and V 2p3/2 confirmed the +5 valence state of vanadium. The peak in the high-resolution XPS spectrum at 529.1 eV (O 1s) was attributed to BiVO_4_-7 lattice oxygen (Olatt) (Figure 3d). The XPS characterization results indicated that BiVO_4_-7 was completely pure.

**Figure 4:**
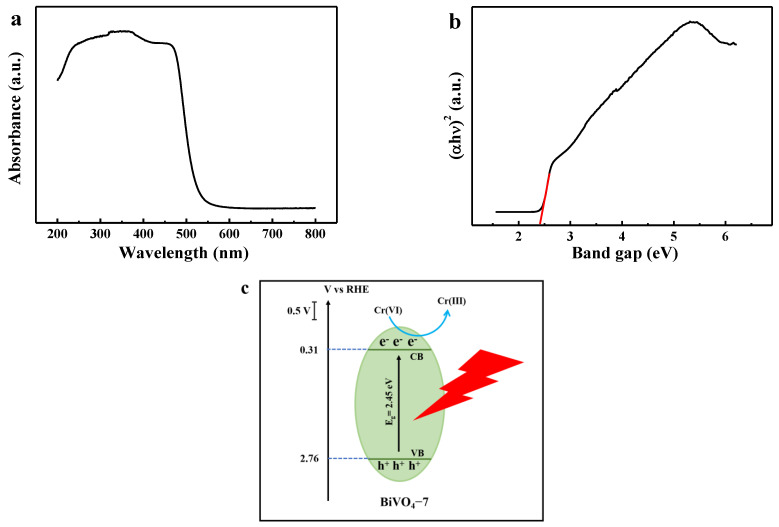
(**a**) UV-Vis diffuse reflectance spectra for BiVO_4_-7; (**b**) plots of (αhν)^2^ vs. the photon energy (hν) for BiVO_4_-7; (**c**) photoelectrochemical mechanism for Cr(VI) detection by BiVO_4_-7.

BiVO_4_-7 showed strong absorption in the visible range, indicating good optical performance (Figure 4a). The BiVO_4_-7 bandgaps were estimated using the formula αhν = A (hν−E_g_)^n^ (E_g_: bandgap; A: a constant; ν: light frequency; α: absorption coefficient). BiVO_4_ had an n of 1/2 [30], and plots of (αhν)^2^ versus the photon energy (hν) were used to estimate the gap energy (E_g_) for BiVO_4_-7 of 2.45 eV (Figure 4b). The optical band structure of BiVO_4_-7 was calculated based on the PEC mechanism. The energies of the conduction band (CB) and valence band (VB) of BiVO_4_-7 were estimated using the following formulae.

E_CB_ = X − E_c_ − 0.5 E_g_ (1)

E_VB_ = E_CB_ + E_g_ (2)

E_c_ denotes the energy of free electrons on the hydrogen scale. This value was approximately 4.5 eV, and the electronegativity (X) of BiVO_4_ was 6.035 eV [31]. The formula presented above was used to calculate the E_CB_ and E_VB_ of BiVO_4_-7 as 0.31 and 2.76 eV, respectively. Under visible light irradiation, BiVO_4_-7 generated hole-electron pairs (h^+^/e^−^) that reduced Cr(VI) to Cr(III) and changed the photocurrent (Figure 4c).

### 3.2. Photoelectrochemical Detection of Cr(VI)

**Figure 5:**
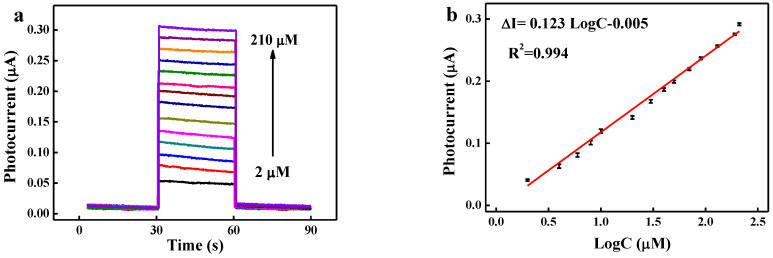
(**a**) Photocurrent response curve and (**b**) corresponding calibration curve for detection of different concentrations of Cr (VI) (2 to 210 μM) using BiVO_4_-7.

An ultrasensitive BiVO_4_-7 PEC sensor was developed to detect Cr(VI). The PEC response of the BiVO_4_-7 sensor to different Cr(VI) concentrations is shown in Figure 5a. The photocurrent increased significantly with the chromium concentration because the reduction of Cr(VI) to Cr(III) accelerated electron transfer. The regression equation for the corresponding logarithmic calibration curve (Figure 5b) was ΔI = −0.005 + 0.123 logc, with a correlation coefficient of 0.994. Here, c represents the Cr(VI) concentration, and ΔI = I − I_0_, where I and I_0_ represent the photocurrent and dark current, respectively. The relation between ΔI and the Cr(VI) concentration was highly linear in the range of 2–210 μM, and the limit of detection (LOD, S/N = 3) was deduced to be 0.01 μM. Compared to previous reports, the LODS of the proposed PEC sensor were 10–200 fold lower and the linear range was wider due to the use of BiVO_4_-7 (Table 1).

### 3.3. Repeatability, Reproducibility, Stability, and Selectivity of the BiVO_4_-7 Sensor

**Figure 6:**
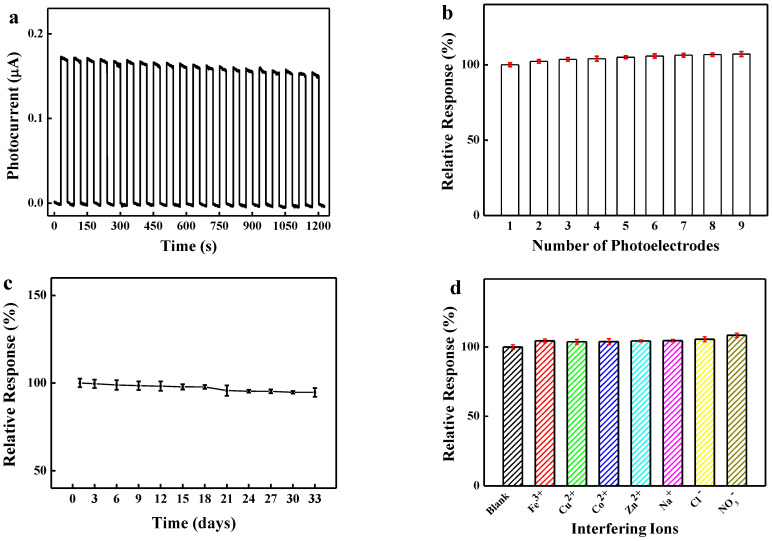
(**a**) The repeatability (short-term stability) for 20 consecutive tests for 50 mM Cr(VI) under a 0.4 V bias voltage; (**b**) reproducibility based on nine parallel photoelectrodes; (**c**) long-term stability, and (**d**) selectivity of the BiVO_4_-7 sensor.

Twenty consecutive on/off switching loops of the light attenuated the photocurrent intensity by less than 10% for 50 μM Cr(VI) and a 0.4 V bias voltage (Figure 6a), demonstrating excellent repeatability for the BiVO_4_-7 PEC sensor. A relative standard deviation (RSD) of 2.24% was determined using nine electrodes to detect 50 μM Cr(VI), showing the high reproducibility of the BiVO_4_-7 PEC sensor (Figure 6b).

In a long-term stability experiment lasting 33 days, 94.6% of the initial photocurrent of BiVO_4_-7 was retained (Figure 6c). A detection specificity experiment was performed by adding a tenfold concentration of interfering ions (Cl^−^, NO_3_^−^, Fe^3+^, Cu^2+^, Co^2+^, Zn^2+^, and Na^+^) to 10 μM hexavalent chromium in an electrolyte. The results demonstrated the photocurrent was retained in the presence of the spiked interfering ions (Figure 6d), indicating that the BiVO_4_-7 PEC sensor had excellent selectivity.

### 3.4. Real Sample Analysis

The BiVO_4_-7 PEC sensor was used to detect Cr(VI) in peanut, rice, soil, and tap water samples to validate the sensor applicability. We spiked real samples with 10 and 100 μM Cr(VI) and obtained good recoveries of 90.3–103% with RSDs less than 8.39% (Table 2). The results indicated that the BiVO_4_-7 PEC sensor could be used for Cr(VI) detection in environmental and food safety monitoring.

## 4. Conclusions

In summary, we synthesized BiVO_4_ (BiVO_4_-7) with a carnation-like morphology by fine pH adjustment without the use of a surfactant. The unique morphology and high specific surface area of the BiVO_4_-7 PEC sensor resulted in high performance for Cr(VI) detection, with a wide linear range of 2–210 μM and a low LOD of 0.01 μM. This PEC sensor exhibited outstanding repeatability (20 times), long-term stability over 33 days, excellent reproducibility, and selectivity. Moreover, the PEC sensor showed excellent accuracy for Cr(VI) detection in peanuts, rice, soil, and tap water, with satisfactory recovery rates of 90.3 to 103.0%. This BiVO_4_-7 based PEC sensor has broad potential in environmental and food safety monitoring.

## Figures and Tables

**Table 1:** Comparison between the performance of the proposed PEC sensor and reported results for Cr(VI) detection.

Materials	Technique	Linear Range (μM)	LOD (μM)	Ref.
GCPF	Fluorescence	0–50	0.22	[32]
GA-AuNPs	Colorimetry	2–20	2.0	[33]
Pd/Ti	DPV	19–100	0.1	[34]
SQDs	Fluorescence	10–120	0.36	[35]
CDs/C_3_N_4_	Fluorescence	2–80	0.39	[36]
CDs@Eu-MOFs	Fluorescence	2–100	0.21	[37]
BiVO_4_-7	PEC	2–210	0.01	This work

GA-AuNPs: gallic acid capped gold nanoparticles; DPV: differential pulse voltammetry; SQDs: sulfur quantum dots; CDs: carbon dots; GCPF: glutaraldehyde cross-linked chitosan polymer fluorophores; CDs@Eu-MOFs: nitrogen and cobalt (II) co-doped carbon dots encapsulated in europium metal–organic frameworks.

**Table 2:** Cr(VI) detection results for a real sample using the PEC sensor (n = 3).

Samples	Original	Added (μM)	Found (μM)	RSD (%)	Recovery (%)	Flame Atomic Absorption Spectrometry (μM)
Peanut	Not found	10	9.90	2.36	99.0	9.93
		100	96.3	3.37	96.3	97.0
Rice	Not found	10	9.41	2.44	94.1	9.45
		100	97.2	2.57	97.2	98.0
Soil	Not found	10	9.26	4.04	92.6	9.30
		100	90.3	8.39	90.3	91.0
Tap water	Not found	10	9.38	1.22	93.8	9.28
		100	103.0	3.87	103.0	101.2

## Supplementary Materials

The following supporting information can be downloaded at: https://www.mdpi.com/article/10.3390/bios12020130/s1, Figure S1. The real picture of the PEC sensor: (A) the detection instrument of the PEC sensor; (B) the three-electrode system. Figure S2. SEM images of (a, b) BiVO_4_-1, (c, d) BiVO_4_-4, (e, f) BiVO_4_-9, and (g, h) BiVO_4_-12.
