# Supplementary material for: Carnation-like Morphology of BiVO4-7 Enables Sensitive Photoelectrochemical Determination of Cr(VI) in the Food and Environment
Source: Biosensors (Basel). 2022 Feb 19;12(2):130. doi: 10.3390/bios12020130 (PMC8870108; doi:10.3390/bios12020130)
Supplement: Supplementary file 1 [file biosensors-12-00130-s001.zip › biosensors-1582631-supplementary.pdf]

biosensors

MDPI

Supplementary Material

Carnation-Like Morphology of $BiVO_4$-7 Enables Sensitive Photoelectrochemical Determination of Cr(VI) in the Food and Environment

Wenqin Wu [1,2,†], Zhao Tan [1,2,3,†], Xiao Chen [1,2,3], Xiaomei Chen [1,2], Ling Cheng [1,2], Huimin Wu [3], Peiwu Li [1,2] and Zhaowei Zhang [1,2,*]

[1] Key Laboratory of Detection for Mycotoxins, Ministry of Agriculture and Rural Affairs, National Reference Lab for Biotoxin Test, Oil Crops Research Institute of the Chinese Academy of Agricultural Sciences, Wuhan 430062, China; wuwenqin@caas.cn (W.W.); 13476831558@163.com (Z.T.); 202021106010763@stu.hubu.edu.cn (X.C.); chenxiaomei_200870@126.com (X.C.); chengling@caas.cn (L.C.); peiwuli@oilcrops.cn (P.L.)
[2] Key Laboratory of Biology and Genetic Improvement of Oil Crops, Ministry of Agriculture and Rural Affairs, Oil Crops Research Institute of the Chinese Academy of Agricultural Sciences, Wuhan 430062, China
[3] College of Chemistry and Chemical Engineering, Hubei University, Wuhan 430062, China; whm267@hubu.edu.cn
* Correspondence: zwzhang@whu.edu.cn; Tel.: +86-27-86711839
† These authors contributed equally to this work.

Citation: Wu, W.; Tan, Z.; Chen, X.; Chen, X.; Cheng, L.; Wu, H.; Li, P.; Zhang, Z. Carnation-Like Morphology of $BiVO_4$-7 Enables Sensitive Photoelectrochemical Determination of Cr(VI) in the Food and Environment. *Biosensors* **2022**, *12*, 130. https://doi.org/10.3390/bios12020130

Received: 19 January 2022
Accepted: 16 February 2022
Published: 19 February 2022

Publisher's Note: MDPI stays neutral with regard to jurisdictional claims in published maps and institutional affiliations.

Copyright: © 2022 by the authors. Licensee MDPI, Basel, Switzerland. This article is an open access article distributed under the terms and conditions of the Creative Commons Attribution (CC BY) license (https://creativecommons.org/licenses/by/4.0/).

S1. Reagents and instruments

The PEC sensor was comprised of a light source and a detection system. The light source was a xenon lamp purchased from Perfect Light Technology Co., Ltd. (PLS-SXE 300, $\lambda \geq 420$ nm, size: $200 \times 370 \times 130$ mm, Beijing, China). The detection system consisted of a CHI 660E electrochemical workstation (CH Instruments Ins., Shanghai, China) and a three-electrode system. The reference electrode was a saturated calomel electrode (SCE), the counter electrode was a Pt wire electrode, and the working electrode was a modified indium tin oxide (ITO) glass electrode. The sensor was immersed in the electrolyte (0.1 M $NaSO_4$) to detect Cr(VI).

X-ray diffraction (XRD, X'Pert PRO MPD, Philips, Holland) on an X'Pert Pro diffractometer with Cu Kα radiation as the X-ray source was used to characterize the crystalline phase composition of $BiVO_4$-X. Scanning electron microscopy (SEM, Gemini 300, Zeiss, Germany) was carried out using a Zeiss GeminiSEM 300 to characterize the morphology of $BiVO_4$-X. X-ray photoelectron spectroscopy (XPS, Thermo ESCALAB 250XI, Thermo Fisher Scientific, USA) was carried out using an ESCALAB 250Xi to identify the surface chemical composition and valence state of the materials. Diffuse reflection spectroscopy (DRS) was performed on the materials using a UV-Vis spectrophotometer (Lambda 750, PerkinElmer, Shanghai, China) with $BaSO_4$ as a reference.

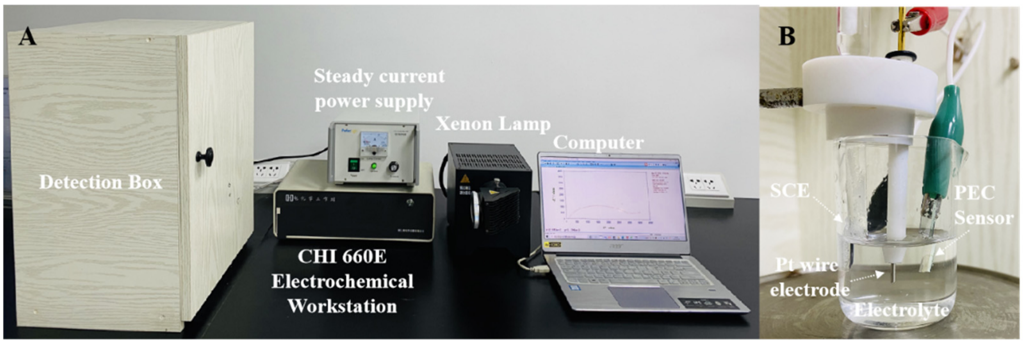

Figure S1. The real picture of the PEC sensor: (**A**) the detection instrument of the PEC sensor; (**B**) the three-electrode system.

S2. Photoelectric Performance Comparison between the Blank ITO Substrates and the $BiVO_4$-7 Modified ITO Sensor in the Electrolyte

The photoelectric performance was compared between the blank ITO substrates and the $BiVO_4$-7 modified ITO sensor by immersed sensor in the electrolyte (0.1 M Na_2SO_4), and an electrochemical workstation was used to record the current signal with or without light. The calculated photocurrent densities generated by a blank ITO substrate (0.172 $nA\cdot cm^{-2}$) are 500 times less than that of the $BiVO_4$ functionalized ITO sensor (86.0 $nA\cdot cm^{-2}$).

S3. SEM Characterization

The morphology of $BiVO_4$-X (X = 1, 4, 9, and 12) was characterized by SEM. Figure S1 shows that under low pH synthesis conditions, the morphology of the material is disordered and uneven. $BiVO_4$-1 (Figure S2a,b) resembles irregular rocks, and $BiVO_4$-4 (Figure S2c,d) has the appearance of wrinkled bark. Under high-pH synthesis conditions, the sheet-like assembled structure of the material collapsed slightly for $BiVO_4$-9 (Figure S2e,f) and severely for $BiVO_4$-12 (Figure S2g,h).

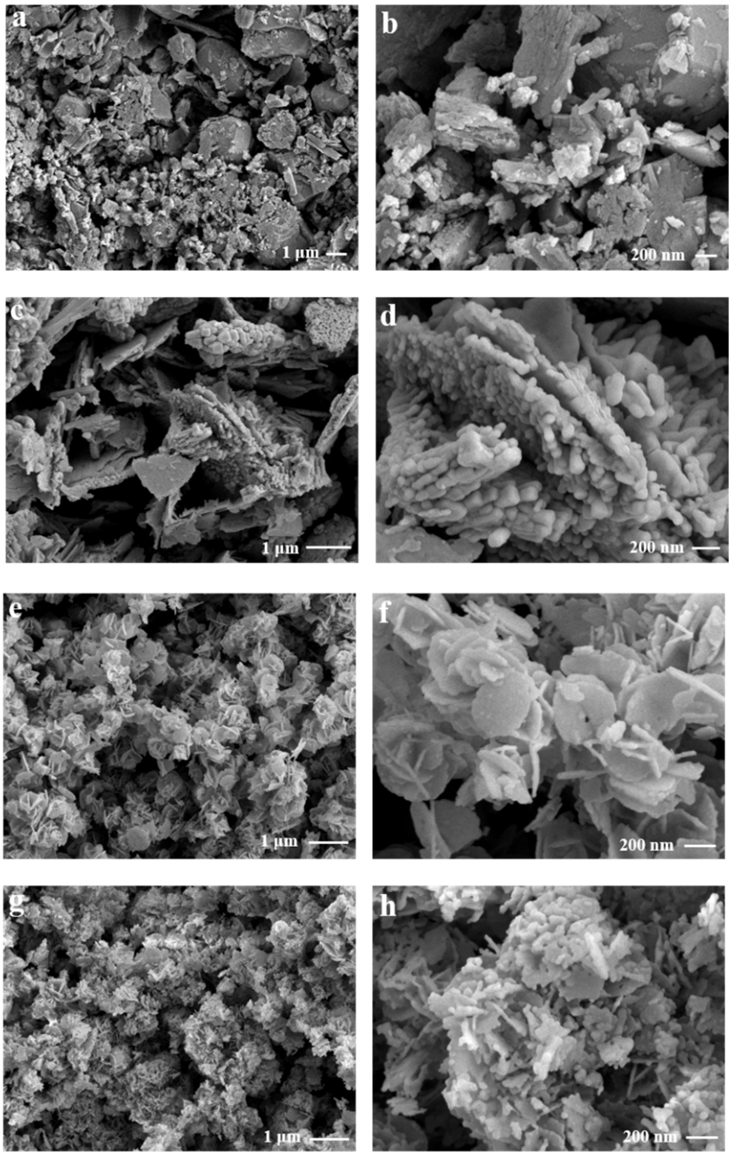

Figure S2. SEM images of (**a**,**b**) $BiVO_4$-1, (**c**,**d**) $BiVO_4$-4, (**e**,**f**) $BiVO_4$-9, and (**g**,**h**) $BiVO_4$-12.
